# Advances in Catalyst Design for β-Lactone Formation via Ring-Expansion Carbonylation

**DOI:** 10.3390/molecules30071399

**Published:** 2025-03-21

**Authors:** Ali Hasnain, Vinothkumar Ganesan, Sungho Yoon

**Affiliations:** Department of Chemistry, Chung-Ang University, Seoul 06974, Republic of Korea; hasnain110@cau.ac.kr (A.H.); vinothcau@cau.ac.kr (V.G.)

**Keywords:** epoxides carbonylation, cobalt carbonyl complexes, heterogeneous catalysis, Friedel–Crafts reaction, β-lactones

## Abstract

Over the past three decades, β-lactones have emerged as valuable intermediates for producing diverse industrial chemicals and biodegradable polymers. The ring-expansion carbonylation (REC) of epoxides has become an atom-economical and direct approach to β-lactone production, leveraging readily available carbon monoxide and epoxides. While homogeneous catalysts, particularly bimetallic [Lewis acid]^+^[Lewis base]^−^-type systems, have demonstrated exceptional activity and selectivity, issues like recycling and separation limit the industrial scalability. Heterogenized catalysts offer advantages such as ease of separation and reusability but suffer from reduced efficiency. Recent advancements in porous polymer-based heterogeneous systems, including immobilized cobaltate anions, address these challenges by combining high surface areas with enhanced catalytic performance. Herein, we explore the evolution of homogeneous to heterogeneous REC catalysts, highlighting emerging porous materials and their potential for scalable β-lactone synthesis. Future directions emphasize overcoming the remaining barriers to establish robust, efficient, and sustainable catalytic processes.

## 1. Introduction

Over the last three decades, β-lactones have become appealing ingredients for the synthesis of a diverse array of industrial chemicals, including but not limited to acrylic acid, β-hydroxy acids, succinic anhydrides, and biodegradable polyester poly(β-hydroxyalkanoates) [1,2,3,4,5,6,7,8]. The noteworthy attributes of β-lactones, characterized by substantial ring strain (22.8 kcal mol^−1^) and two potential sites for nucleophilic attack (C_2_ and C_4_) classify them as ‘privileged’ structures that are capable of undergoing a broad spectrum of transformations [9,10,11,12]. Despite their significance, synthetic routes to β-lactones are confined to challenging and expensive biosynthetic pathways, resulting in limited utilization in the chemical industry. In the case of REC, two major substrates have been introduced, commonly known as epoxides and aziridines [13,14]. Epoxides are easier to synthesize and are more readily available for purchase than aziridines. Several enantiopure catalysts have been developed to synthesize various enantiopure epoxides [14,15,16].

Moreover, the products of epoxide REC, β-lactones, are highly sought after in synthetic chemistry due to their versatility in organic synthesis and their significance in natural products and the synthesis of biologically active compounds [6,17]. These are also well-known monomers to produce biodegradable polyhydroxyalkanoates (PHAs) [7,17,18,19,20]. Additionally, the synthesis of β-lactones, especially in enantiomerically pure form, can only be achieved using catalysts that demonstrate tolerance to diverse functionalities, exhibit high productivity, are straightforward to synthesize, and can be employed with equipment commonly found in most synthetic laboratories. In recent years, the development of the REC of epoxides has emerged as an atom-economic, convenient, and direct method for the efficient production of β-lactones, utilizing the readily available C_1_ source CO, and epoxides [21]. During the previous three decades, substantial advancements have been achieved in improving the activity and selectivity of homogeneous catalysts used in the REC pathway for β-lactone synthesis [22]. The introduction of the ground-breaking work by Coates et al. radically altered the effectiveness of well-defined bimetallic [Lewis acid]^+^[Lewis base]^−^ pair catalysts in the efficient synthesis of β-lactones [23,24]. Despite their homogeneous nature, these catalysts have progressed toward commercialization, overcoming challenges associated with laborious product separation and catalyst recycling. To address the issues of product separation and recycling, there is a compelling need for a heterogeneous catalytic process.

Although classical heterogeneous catalysts utilized in industries offer advantages such as facile separation, recycling, and practical applicability in continuous flow processes, they often suffer from drawbacks including reduced activity, selectivity, and the requirement for harsh reaction conditions such as high temperature and pressure. Consequently, developing a well-defined heterogeneous catalyst that facilitates the rapid and selective REC of epoxides has become a paramount challenge. In the recent past, reports on porous heterogeneous REC processes have begun to surface, challenging the viability of heterogeneous processes for β-lactone production [25]. The potential for the post-synthetic immobilization of cobaltate anions through metathesis reactions involving labile halogen ligands solidifies the concept of generating heterogenized [Lewis acid]^+^[Lewis base]^−^ pair catalysts. However, ongoing research and developments in realizing this heterogeneous strategy remain vibrant, particularly with the advent of porous polymeric materials featuring high surface area solid catalysts [26,27]. In this context, recent reports detailing the potential application of porous polymeric materials for the heterogeneous REC of epoxides are comprehensively reviewed for the first time. Furthermore, to provide a comprehensive understanding of the concepts surrounding REC and the reaction mechanisms, developments in the contemporary period are briefly summarized, complementing previous extensive reviews on the subject [28].

## 2. Evolution of Ring-Expansion Carbonylation of Epoxides

**Scheme 1:**
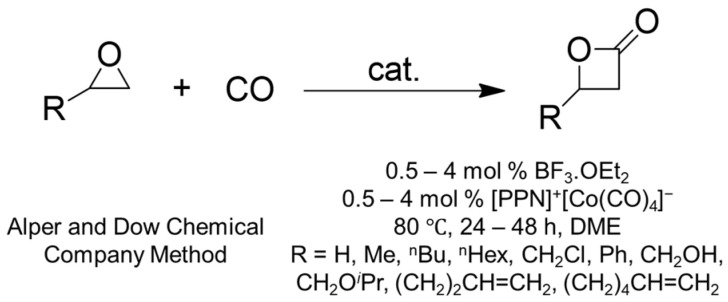
Alper method for the carbonylation of epoxides to produce β-lactones [2].

In 1963, Richard Heck started working with isobutylene oxide carbonylation, giving the nucleophilic attack by a cobalt tetracarbonyl anion [Co(CO)_4_]^−^ at the less sterically hindered position. After the insertion of carbon monoxide (CO), an acyl cobalt species forms, which is cleaved by methanol to form methyl 3-methyl-3-hydroxybutyrate selectively. However, with the halogenated carbonylation process requiring a higher loading of sodium cobalt carbonyl (NaCo(CO)_4_), this discovery was an important foundation of cobalt chemistry in the REC of epoxides [1]. After this study, the appeal to the carbonylation system began with the same substituents in the 1994 patent by Drent and Kragtwijk [29], who developed a Co_2_(CO)_8_/3-hydroxypyridine in situ catalytic system for the carbonylation of isobutylene oxide regioselectively, achieving 60% yields of β-lactones or β-hydroxyesters with minimal polyester formation. Additionally, the authors emphasized that the reaction conditions significantly influenced the product distribution, with elevated temperatures possibly pyrolyzing, pressure, or extended reaction times favoring the conversion of β-lactones into polyesters [30]. In 2001, Alper and the Dow Chemical Company revisited Drent and Kragtwijk β-lactone synthesis but achieved only 15% β-lactone and 75% polyester, significantly lower than the reported 96% conversion and 90% selectivity (Scheme 1).

However, they demonstrated that a [PPN]^+^[Co(CO)_4_]^−^ ([PPN]^+^ = bis(triphenylphosphine)iminium) Lewis acid–base pair, combined with neutral species such as BF_3_·Et_2_O or SnCl_4_, catalyzed the carbonylation of various epoxides to β-lactones with high yields (60–90%) under 62 bar CO pressure (Scheme 1) [2]. The in situ generated catalyst from PPNCl/Co_2_(CO)_8_ and BF_3_·OEt_2_ requires the Lewis acid for activation and a nucleophilic metal carbonyl, with B(C_6_F_5_)_3_ producing a more active but less selective system that affects the stereochemistry in β-lactone formation from epoxides. Despite its pioneering selectivity in epoxide REC, the catalyst’s high loading, long reaction times (24–48 h), and harsh conditions highlight the need for more efficient alternatives.

**Figure 1:**
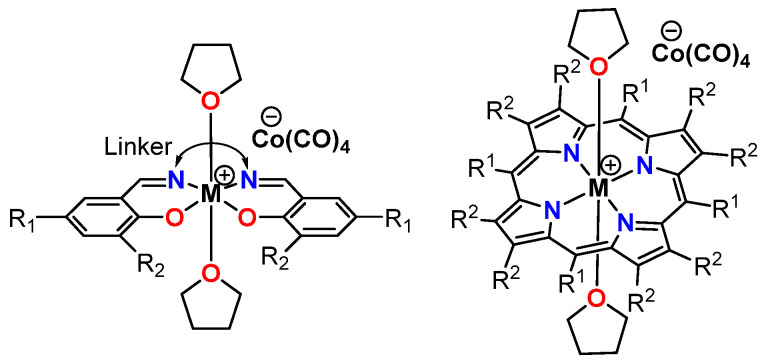
Fundamental well-defined catalyst frameworks (**left**—salph, and **right**—porphyrin) for the REC to β-lactone formation.

In 2002, Coates et al. developed a highly efficient salph-based catalyst, [(salph)Al(THF)_2_]^+^[Co(CO)_4_]^−^, (salph = *N*,*N*′-o-phenylenebis(3,5-di-tert-butylsalicylideneimine), THF = tetrahydrofuran), for the REC of epoxides to β-lactones, enabling the scalable synthesis of poly[(*R*)-β-hydroxybutyrate)] (R-PHB) [24]. The catalyst effectively combines a Lewis acidic aluminum cation with a nucleophilic cobaltate anion, achieving selective carbonylation under mild conditions. Their work represents a significant breakthrough in the catalytic conversion of readily accessible epoxides and CO to β-lactone using a well-defined catalyst and advancing the production of biodegradable polymers [24,31]. Various catalytic systems with the general formula [Lewis acid]^+^[Co(CO)_4_]^−^ have also been developed, exhibiting tunable reactivity and selectivity based on the metal and ligand used in Figure 1.

In the case of shelf-life, aluminum salen catalysts (Table 1, entry 1) have been proven to be remarkably effective in REC reaction. The aluminum salen complex was fully characterized by NMR, IR, and X-ray crystallography, which identified that the THF was axially bound and capable of coordinating with cyclic ether. This catalyst was used to carbonylate 1-butene oxide to the highest yield in 2.5 h under 60 bar CO pressure at 50 °C in 1 mol % Co loading without any solvent [24]. Another report described the *trans*-disubstituted epoxides REC with excellent regioselectivites. The catalytic framework was redesigned from the salen-type to a salalen-type with the addition of a pyridine donor ligand and the fine-tuned steric size of the aryl group, which led to an improvement in the REC activity at room temperature (Table 1, entries 2 and 3) [32]. To advance the field of epoxide carbonylation, Coates and co-workers developed controlled and contrasteric catalysts for isobutylene oxide to pivalolactone and poly(pivalolactone), which promoted the SN1 type to produce the α,α-disubstituted lactone in >99% regioselectivity (Table 1, entry 4) [33].

To install this DABN-based aluminum complex with an iminonaphthol moiety, the catalyst prevents the nucleophile from attacking the less sterically hindered position, which can further stabilize the partial positive charge at the more congested epoxide site by noncovalent interaction. In addition, the salicylaldehyde bearing the sterically hindered mesityl substituent, the catalyst gave good enantioenriched lactone from racemic *cis*-epoxides (Table 1, entry 5) [34]. In this salen-based catalyst, the ligand cis-α coordination around the Lewis acidic metal ion was attributed to the enantioenrichment system. In another study, Coates and co-workers investigated alkyl epoxide with various substituent patterns incorporating cobalt carbonyl compounds, and Lewis acids coordinated by salen-based chromium scaffolds were successfully converted into β-lactones under only 1 atm of CO at ambient temperature (Table 1, entry 6) [35].

In 2007, a porphyrin-based bimetallic catalyst [(ClTPP)Al(THF)_2_]^+^[Co(CO)_4_]^−^ (ClTPP = meso-tetra(4-chlorophenyl)porphyrinato) was introduced for the double carbonylation of epoxides to a β-lactone in the first stage, and then the β-lactone was subsequently converted to a succinic anhydride in excellent yield (Table 1, entry 7) [4]. These two stages of transformation were studied by in situ IR spectroscopy, which revealed that both carbonylations occurred subsequently without overlapping, and β-lactone carbonylation proceeded after all the epoxide was consumed.

**Scheme 2:**
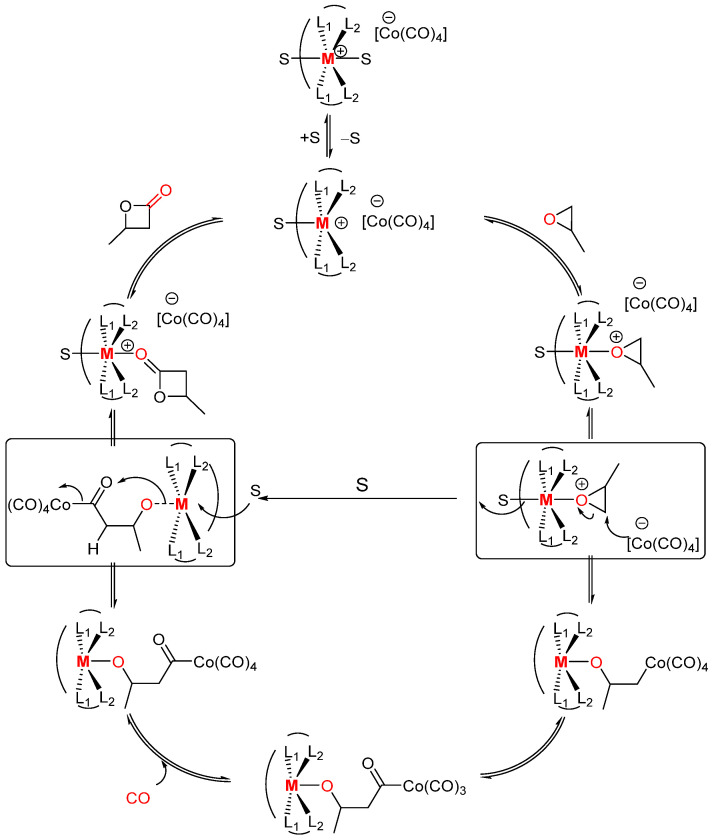
Proposed mechanism for the carbonylation of epoxides using catalysts of the form [Lewis acid]^+^[Co(CO)_4_]^−^, M = Al^3+^ or Cr^3+^, L_1_, L_2_ = N for porphyrin, L_1_ = N, L_2_ = O for salph, S = solvent [36].

Shortly afterward, the same group reported well-defined [Co(CO)_4_]^−^-based bimetallic catalysts [(TPP)Cr(THF)_2_]^+^[Co(CO)_4_]^−^, [(OEP)Cr(THF)_2_]^+^[Co(CO)_4_]^−^ (OEP = octaethylporphyrinato), and [Cp_2_Ti(THF)_2_]^+^[Co(CO)_4_]^−^ with high catalytic activity and regioselectivity under the key concept on the proposed reaction mechanism (Scheme 2). All complexes were found to catalyze the REC of a broad array of epoxides under the conditions of relatively low CO pressure, low catalyst loading, and low reaction temperature. The porphyrin-based ligand system and chromium metal in the Lewis acid part of the catalyst identified the strong effect with respect to the catalytic activity and wide scope of applicable substrates.

**Figure 2:**
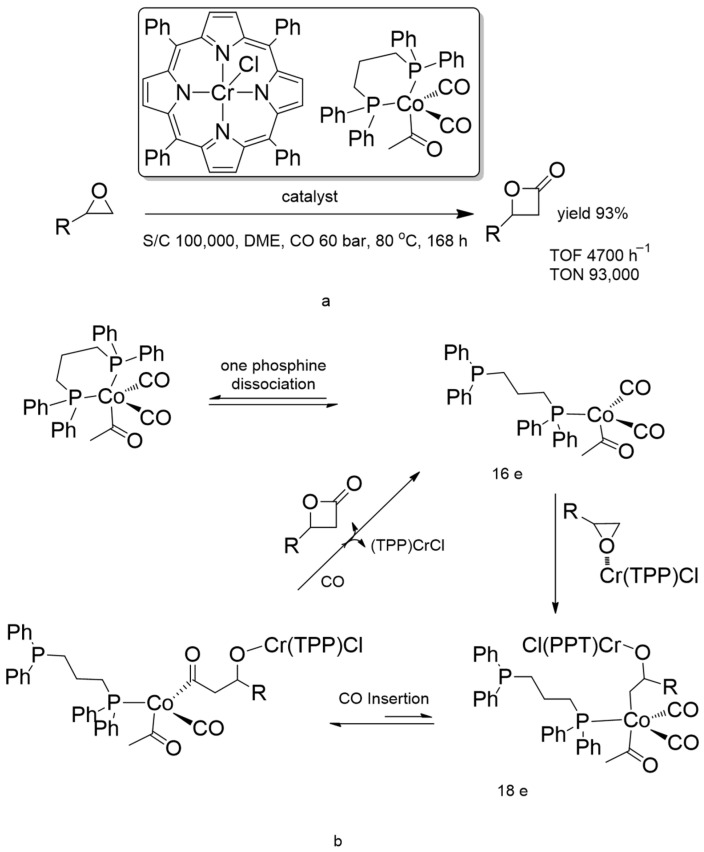
Epoxide (C_6_) carbonylation reaction (**a**) and proposed mechanism (**b**) [3].

In general, the Cr-Co catalyst system was superior to the Al-Co and Ti-Co systems due to higher yield and lower catalyst loading (Table 1, entries 8 and 9) [37,38]. For example, regarding the carbonylation of epoxides to β-lactones, the catalyst [(OEP)Cr(THF)_2_]^+^[Co(CO)_4_]^−^ gave excellent TONs (turnover numbers) up to 10,000 and TOFs (turnover frequencies) up to 1670 h^−1^ with regioselective CO insertion between the oxygen and the less substituted carbon of the epoxides [38]. Yoon and co-workers reported their studies on the REC of epoxides (Hexene oxide) with the stable catalysts [(acetyl)Co(CO)_2_dppp] (dppp = 1,3-bis(diphenylphosphino)propane) and [(TPP)CrCl] to give β-lactone in 93% yields and the highest TONs up to 93,000 and TOFs up to 4700 h^−1^ with 60 bar of CO insertion (Figure 2) [3].

Interestingly, the coordinative unsaturated acetyl Co(CO)_2_ species was formed through one phosphine dissociation from the cobalt center. It was anticipated that the formed stable acetyl Co(CO)_2_ species led to the attack on the methylene carbon, or potentially the methine carbon, in the [(TPP)CrCl] activated epoxide. In this mode of reactivity, an 18-electron ring opened intermediate was generated, and a reaction intermediate en route to form the next species after CO insertion, which then eventually afforded the ring closure, giving β-lactone in a 93% yield (Figure 2).

Gallium porphyrin and tin porphyrin have also been applied as Lewis acid catalysts for the REC of epoxides in good yields (Table 1, entries 10 and 11) [39,40]. Although gallium porphyrin shows a strong oxophilic Lewis acidic nature, the larger radius and longer bond length of Ga(III)-O may assist to dissociate faster to produce β-lactones, so we believe that specific Lewis acid and nucleophilic metal carbonyl combinations could also prove to be more effective candidates.

**Table 1:** Salph and porphyrin catalytic substituent nature and β-lactone production yield.

Entry	Metal	Salph Catalysts	Yield (%)	Ref.
1	Al^3+^	Linker = 1,2-C_6_H_4_, and R_1_, R_2_ = *^t^*Bu	99	[24]
2	Al^3+^	Linker = (R,R)-1,2-C_6_H_10_,with amine and R_1,_ R_2_ = *^t^*Bu	95	[32]
3	Al^3+^	R_1_ = Me, R_2_ = 2,6-Me2-4-*^t^*BuPh (Ar^3^)	95	[32]
4	Al^3+^	Linker = 2,2′-diamino-1,1′-binaphthalene (DABN) and R_1_, R_2_ = Ar^4^	99	[33]
5	Al^3+^	Atropisomeric DABN unit and R_1_ = Me, R_2_ = Mesityl	90	[34]
6	Cr^3+^	Linker = 1,2-C_6_H_4_ and R_1_, R_2_ = *^t^*Bu	98	[35]
		**Porphyrin Catalysts**		
7	Al^3+^	R_1_ = *p*-Cl-C_6_H_4_ and R_2_ = H	90	[4]
8	Cr^3+^	R_1_ = Ph and R_2_ = H	99	[37]
9	Cr^3+^	R_1_ = H and R_2_ = Et	99	[38]
10	Ga^3+^	R_1_ = Ph and R_2_ = H	95	[39]
11	Sn^4+^	R_1_ = 4-chlorophenyl and R_2_ = H	99	[40]

Structural elucidation via single-crystal X-ray crystallography confirmed the formation of frustrated ionic pairs, including salph- and porphyrin-based catalysts, with a pseudo-octahedral cationic complex Lewis acid and a tetrahedral [Co(CO)_4_]^−^ anion as a base pair. In the Coates bimetallic catalytic system, the [Co(CO)_4_]^−^ anion is the key active species responsible for cleaving Co_2_(CO)_8_ or its derivatives. To address the challenges associated with sensitive cobalt carbonyl species, unstable low-valent salts, the in situ reduction of divalent cobalt (II) salts has been developed, enabling efficient epoxide carbonylation with 91% β-lactone yields and 97% selectivity, thus eliminating the need for cobalt carbonyl anions [41].

## 3. Mechanism of Ring-Expansion Carbonylation

Mechanistic studies by Coates and co-workers revealed that the carbonylation of heterocycles is driven by the SN2-type mechanism, where the salen- and porphyrin-based cationic metallic complexes form active species by in situ exploitation (Scheme 2) [4,36]. These structured complexes, with the general formula [Lewis acid]^+^[Co(CO)_4_]^−^, have shown optimized reactivity for classified substrates, which can be distinguished into *cis*-, *trans*-, terminal-, and 2,2-disubstituted epoxides [32,42,43]. Some studies that applied the salen complex system represented changes in stereochemistry such as *cis*- and *trans*-epoxides that were converted to *trans*- and *cis*-β-lactones accordingly [23,44,45]. These stereochemical changes resulted from the inversion of a stereocenter due to nucleophilic attack of the cobalt tetracarbonyl (Co(CO)_4_) anionic species (Scheme 2). The cobalt-alkyl species that forms undergoes the migratory insertion of CO, forming a cobalt-acyl complex that acts as the resting state of the catalytic cycle. Furthermore, the mechanism of the catalytic cycle occurs in these major steps: substrate activation, nucleophilic ring opening, CO insertion, CO uptake, Lewis base ring-closure, and lactone dissociation [46]. Rieger and coworkers also explained the density functional theory (DFT) calculations, which provide insights into the difficult case of catalytic mechanism under the action ligand (e.g., salph) [22].

**Figure 3:**
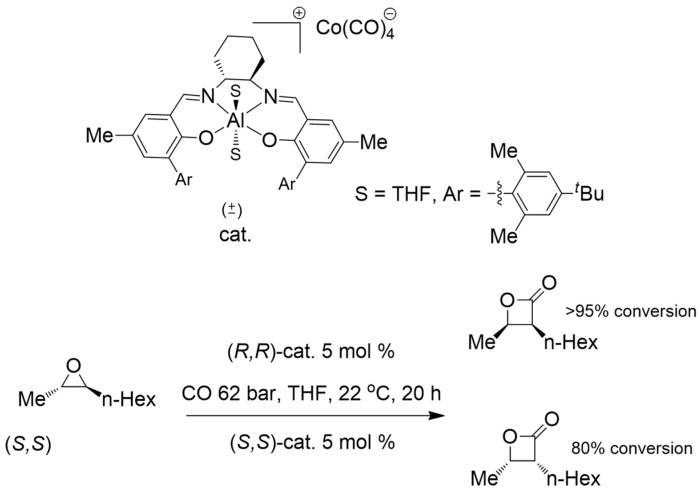
Regioselective carbonylation of the enantioenriched epoxide with the enantiomer catalyst [32].

In addition, regioselective carbonylations of racemic *trans*-epoxides were optimized by using a salen-based complex, as shown in Figure 3 [32]. This reaction resulted when the salen-based catalyst system enantiomer carbonylated enantioenriched epoxides. For instance, complex (*S*,*S*-cat.) showed reduced reactivity toward enantioenriched epoxide. In contrast, both aspects were significantly enhanced when the (*R*,*R*-cat.) system reacted with the same epoxide, obtaining an enantioenriched product with a high yield (Figure 3). These results indicate that the (*R*,*R*-cat.) system has a propensity to generate similar pairs with (*S*,*S*)-*trans*-epoxides.

## 4. Ring-Expansive Carbonylation Epoxides by Heterogeneous Catalysts

While homogeneous catalysts exhibit remarkable efficiency in epoxide carbonylation, their industrial application poses significant challenges due to complications in product separation and the recyclability of the expensive catalyst. These issues hinder the economic and environmental sustainability of the process on a large scale. Heterogeneous catalysts have been developed based on the proposed carbonylation catalytic cycle utilizing [Lewis acid]^+^[Co(CO)_4_]^−^-type catalysts. The crucial involvement of cobaltate species in epoxide ring opening, the migratory insertion of CO between the cobalt-alkyl bond, and the uptake of CO, followed by leaving the Co-Acyl bond to regenerate the active species, forms the basis of the catalytic cycles. Although it was speculated that other nucleophilic metal carbonyls in combination with suitable Lewis acids could be effective for this carbonylation, no alternative nucleophilic metal carbonyl has been reported to date for this specific role. Given the irreplaceable role of the cobaltate anion, it remains unchanged or replaced only with similar frustrated Lewis acid–base interactions in the final catalysts.

**Figure 4:**
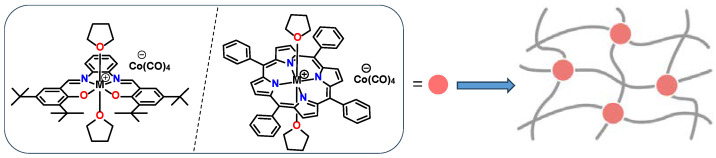
General representation of the direct heterogenization of the active homogeneous ring-expansion carbonylation catalyst system (the red dot represents either Salen-type [Lewis acid]^+^[Co(CO)_4_]^−^ or Porphyrin-based [Lewis acid]^+^[Co(CO)_4_]^−^).

The design of all of the reported heterogeneous catalysts involves alterations only in the Lewis acidic part. Two primary strategies for catalyst heterogenization are the direct heterogenization method and modification of the Lewis acidic part to mimic the pseudo-octahedral Lewis acidic metal center. In the direct heterogenization method, ligands of homogeneous catalysts are knitted together and then metallated to obtain metallated porous organic polymers (POPs). Alternatively, pre-final homogeneous catalysts are directly knitted or crosslinked with suitable linkers to generate POP materials. In this process, labile chloride ions are exchanged with cobaltate anions to create typical [Lewis acid]^+^[Co(CO)_4_]^−^ type active ion pairs (Figure 4). Another approach involves constructing or immobilizing the Lewis acidic part on existing porous support materials in a pre-final form, followed by exchanging with cobaltate anions to obtain the final porous polymeric catalyst of the Lewis acid–base pair type [47,48,49,50].

## 5. Salphen-Type POP Catalysts

**Figure 5:**
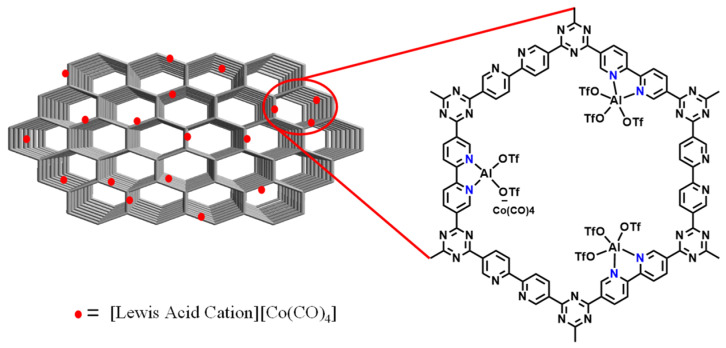
Structural demonstration of CTF supported [bpy-CTF-Al(OTf)_2_]^+^[Co(CO)_4_]^−^ for the carbonylation of PO to β-lactone [25].

Yoon et al. developed a recyclable heterogeneous catalyst, [bpy-CTF-Al(OTf)_2_]^+^[Co(CO)_4_]^−^, using a covalent triazine framework (CTF) with bipyridine ligands to anchor aluminum triflate (Al(OTf)_3_), emulating the metal-N_2_O_2_ ligation of salphen-type catalysts (Figure 5). This anchoring facilitates the metathesis exchange of one triflate ion with a cobaltate anion, creating an Al-N_2_O_2_ Lewis acidic center paired with [Co(CO)_4_]^−^ for the efficient REC of propylene oxide (PO) to β-lactone (Table 2, entry 1) [25]. The catalyst’s porous structure exhibited a specific surface area of 684 m^2^ g^−1^ and a pore volume of 0.4 cm^3^ g^−1^. X-ray photoelectron spectroscopy (XPS) confirmed the successful immobilization of the catalytic species on the CTF support. Under reaction conditions of 50 °C and 60 bar CO pressure, the catalyst achieved over 99% conversion of PO with a 90% selectivity for β-lactone and minimal acetone as a by-product. Additionally, hot-filtration tests confirmed that the catalyst remained immobilized, and recycling studies showed that any decrease in activity and selectivity due to cobalt leaching could be mitigated by regenerating the catalyst with K[Co(CO)_4_]. Although the catalyst exhibited slightly lower activity compared with the homogeneous analogs, this work represents a significant advancement in the heterogenization of homogeneous catalysts, highlighting its potential for industrial applications [25,31].

**Figure 6:**
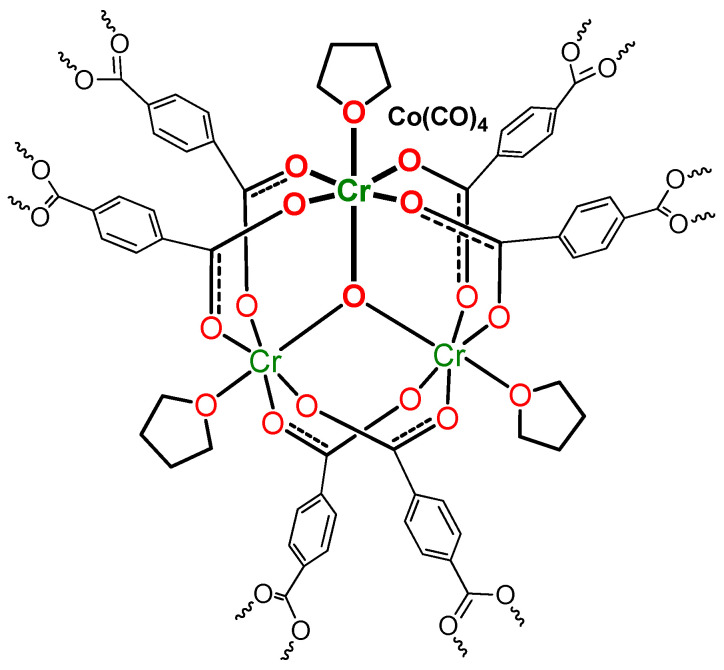
Structural elucidation of Co(CO)_4_⊂Cr-MIL-101 for the REC of epoxides to β-lactone [48].

In 2017, Roman-Leshkov and colleagues introduced a groundbreaking heterogeneous catalyst, [Co(CO)_4_]^−^, incorporated into a Cr(III)-based metal-organic framework (MOF) called Cr-MIL-101. The catalyst excelled in epoxide carbonylation due to its design, which allowed for the efficient post-synthetic exchange of fluoride ions with [Co(CO)_4_]^−^, creating a composite catalyst, Co(CO)_4_⊂Cr-MIL-101, that mimicked the behavior of [Lewis acid]^+^[Co(CO)_4_]^−^ systems (Figure 6). The removal of THF solvent molecules from the Cr(III) sites created Lewis acidic sites that effectively activated epoxides. Remarkably, the [Co(CO)_4_]^−^ species remained stably embedded within the framework, preventing leaching and ensuring sustained catalytic performance. The catalyst demonstrated impressive activity in the carbonylation of 1,2-epoxyhexane, with a site time yield (STY) of 34 h^−1^, making it the most active heterogeneous epoxide carbonylation catalyst reported (Table 2, entry 2) [48]. Its performance was on par with active homogeneous catalysts, offering a broad substrate scope and solvent-dependent behavior akin to homogeneous systems [50].

**Scheme 3:**
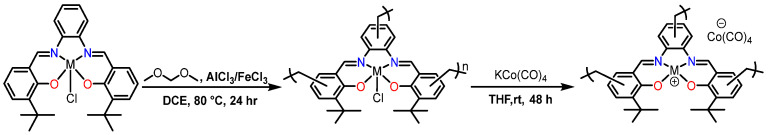
Synthetic representation for the porous salphenM(III)Cl POP and cobaltate exchanged POP [SalphM^+^][Co(CO)_4_]^−^, where M = Al or Cr [51].

In parallel, the synthesis of POPs via the Friedel−Crafts reaction (FCR) catalyzed by Lewis acidic AlCl_3_ or FeCl_3_ has garnered considerable attention. The strength of this approach lies in its simplicity and efficacy. Recently, the FCR method has proven valuable for generating robust porous organic/organometallic polymeric material networks. This method circumvents the need for monomers with specific polymerizable functionalities, thereby avoiding intricate synthetic routes. The approach represents a significant advancement in the synthesis of durable and efficient catalytic materials [49]. The FCR method offers a cost-effective and scalable approach for producing highly porous, resilient networks. Using this technique, homogeneous Schiff base-ligated salphen aluminum and chromium catalysts were successfully heterogenized by covalently linking aromatic rings via the FCR (Scheme 3) [51].

The ^1^H NMR analysis revealed that crosslinking occurred at the *p*-position of the phenolic moiety, forming porous polymeric materials with BET surface areas of 140 and 300 m^2^ g^−1^ for the aluminum and chromium complexes, respectively. Cobaltate anions were incorporated post-synthetically, enabling the efficient REC of epoxides to β-lactones. The heterogenized catalysts demonstrated comparable activity to their homogeneous counterparts, with the chromium catalyst showing >99% conversion and 90% selectivity (Table 2, entries 3 and 4) [51]. Notably, the chromium catalyst was recyclable, maintaining activity over several cycles with regeneration.

## 6. Porphyrin-Based POP Catalysts

**Scheme 4:**
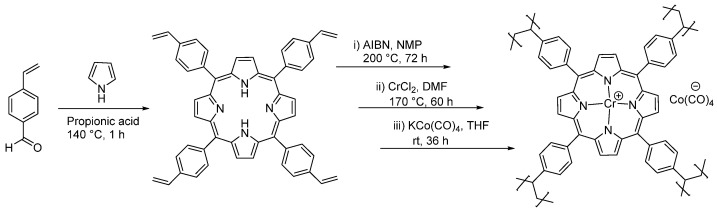
Schematic view of [Cr-metallated PPP]^+^[Co(CO)_4_]^−^ synthesis for the carbonylation of PO to β-lactone [52].

Motivated by the structural robustness inherent in porous polymers, coupled with their hierarchical porosity and drawing inspiration from the superior catalytic prowess exhibited by homogeneous porphyrin-based catalysts in the realm of the ring-expansion carbonylation of epoxides, Yoon et al. conceived and crafted a porous polymeric material incorporating porphyrin moieties (Scheme 4). This material was synthesized through the polymerization of a phenylporphyrin monomer containing vinyl groups, resulting in the formation of a highly porous porphyrin polymer (PPP) characterized by a BET surface area of 1110 m^2^ g^−1^ and a total pore volume of 1.3 cm^3^ g^−1^ [52]. The resultant PPP possessed a remarkable binding affinity for catalytically active species, facilitated by its outstanding hierarchical porous structure, which ensured high accessibility by the reactants. Metallation of the PPP with Cr^3+^ yielded a heterogeneous (PPP)Cr(III)Cl material, akin to the homogeneous tetraphenylporphyrinCr(III)Cl coordination complex, featuring a BET surface area of 877 m^2^ g^−1^. The metallated porous material, (PPP)Cr(III)Cl, underwent a comprehensive characterization process, following which the labile Cl^−^ anions were efficiently exchanged with Co(CO)_4_^−^ anions. This transformation was achieved by treating the material with an excess of KCo(CO)_4_ in a dry THF medium, resulting in the formation of a cobaltate-functionalized [Lewis acid]^+^[Co(CO)_4_]^−^-type heterogeneous carbonylation catalyst obtained in excellent yield.

The heterogeneous [(PPP)Cr]^+^[Co(CO)_4_]^−^ catalyst exhibited comparable PO carbonylation activity to its homogeneous counterpart, [(TPP)Cr(THF)_2_]^+^[Co(CO)_4_]^−^, achieving a 41% conversion and >99% selectivity toward β-lactone. Operated at 60 °C for 20 h under 60 bar CO pressure, with a substrate-to-catalyst ratio of 1000, the STY for β-lactone reached 21 h^−1^ in weakly coordinating dimethoxyethane (DME) solvent. This STY surpassed previous reports on CTF-based heterogeneous catalysts [25]. Impressively, the catalyst demonstrated robust recyclability for up to three cycles, maintaining a yield greater than 95%. Reduced conversion to 90% in the fourth cycle was attributed to cobalt leaching, and catalyst regeneration with KCo(CO)_4_ restored the activity back to 95% (Table 2, entry 5) [52].

Continuing their research, the Yoon group introduced a synthetically facile FCR for the direct heterogenization of a homogeneous carbonylation catalyst [37]. A strategic advantage of employing FCR for POP construction lies in circumventing the necessity for special polymerization functional groups, thereby avoiding a cumbersome step to attach a polymerizable functional group to the monomeric unit. In the direct heterogenization of tetraphenylporphyrin chromium chloride, the knitting strategy utilizes dimethoxymethane (DMM) as a crosslinker, connecting the phenyl rings of tetraphenylporphyrins through a facile FeCl_3_-catalyzed FCR. The pre-metallated monomer, TPPCr(III)Cl, confirms the preservation of the intact Lewis acidic Cr^3+^ center in the resulting polymeric material [53].

**Scheme 5:**
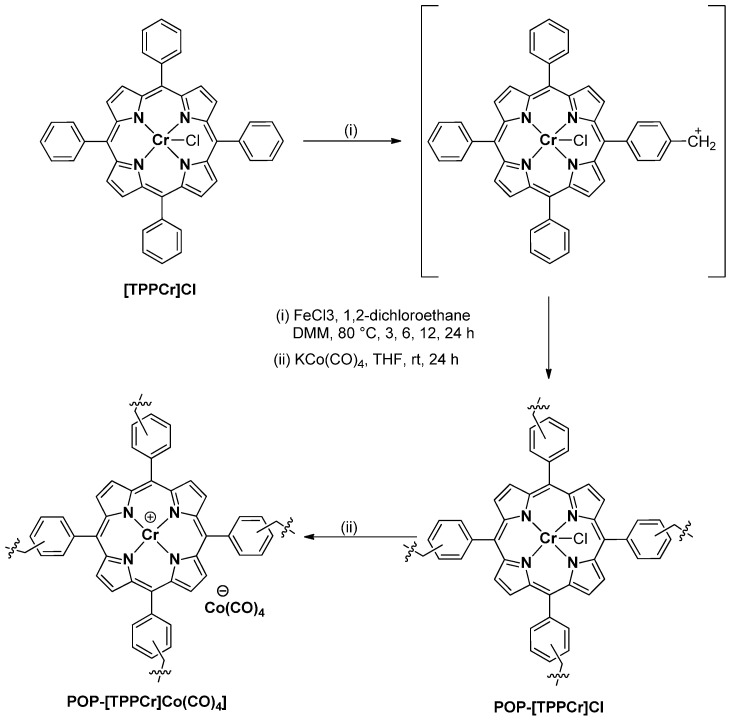
Synthetic steps for the preparation of the POP-[TPPCr]^+^[Co(CO)_4_]^−^ catalyst [53].

Furthermore, the study delved into the correlation between catalyst reactivity and the degree of heterogeneity for the first time, unraveling the intricate relationship between heterogenization and reactivity. Monitoring the FCR progress by UV–Visible spectroscopy over varying time intervals revealed the Soret band shift and the formation of porphyrin methyl cation in the near-IR region, providing insights into the intermediate carbocation during the FCR. Different time intervals of FCR catalyzed heterogenization, namely 3 h, 6 h, 12 h, and was fully heterogenized at 24 h, resulting in varying degrees of polymerized pre-catalyst. Incorporating [Co(CO)_4_]^−^ anions generated different degrees of polymerized catalysts, denoted as POP-[TPPCr][Co(CO)_4_]-3h, POP-[TPPCr] [Co(CO)_4_]-6h, POP-[TPPCr][Co(CO)_4_]-12h, and POP-[TPPCr][Co(CO)_4_]-24h, respectively. This systematic approach not only advances the field of heterogeneous catalysis, but also showcases the strategic integration of synthetic methodologies to tailor catalysts for enhanced performance and versatility (Scheme 5).

In addition, the catalyst underwent comprehensive characterization to elucidate its compositional, porous, morphological, and surface structure before conducting REC using PO as the substrate (2400:1 ratio) in DME at 60 °C under 60 bar CO pressure (refer to Table 2, entries 6–9) [53]. The 3h heterogenized catalyst exhibited a 92% conversion with an initial STY of 400 h^−1^, followed by 75% conversion and an initial STY of 300 h^−1^, 56% conversion with an initial STY of 180 h^−1^, and 43% conversion with an initial STY of 120 h^−1^ for the catalysts POP-[TPPCr][Co(CO)_4_]-3h, POP-[TPPCr][Co(CO)_4_]-6h, POP-[TPPCr][Co(CO)_4_]-12h, and POP-[TPPCr][Co(CO)_4_]-24h, respectively. A detailed study on recyclability revealed that partially heterogenized catalysts were extensively dispersed in the DME solution, exhibiting a comparable REC rate to a homogeneous catalyst but with poor recyclability. In contrast, the completely heterogenized catalyst POP-[TPPCr][Co(CO)_4_]-24h demonstrated high activity, selectivity, and durability during recycling [37,53]. In comparison to the commercially available TPPCr(III)Cl, the fully heterogenized catalyst POP-[TPPCr][Co(CO)_4_]-24h emerged as a promising candidate for the large-scale production of β-lactones.

**Scheme 6:**
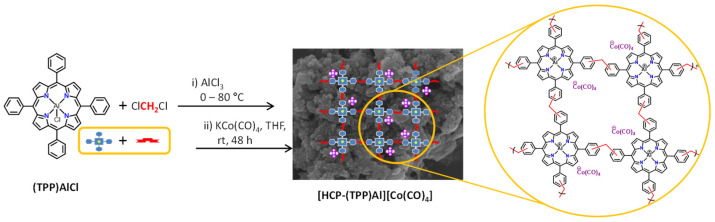
Structural representation of the synthesis of the [HCP-(TPP)Al]^+^ [Co(CO)_4_]^−^ catalyst (the blue and red(line) symbol represents (TPP)AlCl and the methylene linker respectively) [54].

In a subsequent study by Yoon et al., a highly active homogeneous Al(III) tetracarbonylcobaltate bimetallic catalyst was heterogenized using a hyper-crosslinking strategy through methylene bridges by AlCl_3_ catalyzed FCR [54]. The innovative approach knits together aromatic rings of homogeneous catalysts, creating a robust, highly porous solid catalyst with a surface area of 1100 m^2^ g^−1^. This exceptional porosity offers dual benefits by enabling effective mass transfer into the catalyst and indicating ample space for further functionalization. The resulting hyper-crosslinked polymer, HCP-(TPP)AlCl, was further functionalized through a metathesis reaction with KCo(CO)_4_, yielding a [Lewis acid]^+^[Co(CO)_4_]^−^-type catalyst (Scheme 6).

The catalyst demonstrated exceptional performance in the REC of epoxides, achieving high selectivity (>99%) toward corresponding β-lactones and the highest reported initial STY of 360 h^−1^ for 1,2-epoxyhexane (Table 2, entry 10) [54]. The catalyst also exhibited broad substrate tolerance and excellent recyclability, maintaining activity over multiple cycles with regeneration.

## 7. Phthalocyanine-Based POP Catalysts

To extend the repertoire of ring-expansion carbonylation chemistry beyond conventional salicylaldehyde/salophen Schiff base and porphyrinoid ligated complexes, phthalocyanine (Pc), an analog of porphyrin, has been introduced as a novel catalytic system for both the mono and double ring-expanding carbonylation of epoxides [57]. In this investigation, the catalyst system comprised in situ generated [Lewis acid]^+^[Co(CO)_4_]^−^ species formed from commercially available components, namely aluminum(III) phthalocyanine chloride (AlPcCl) and dicobaltoctacarbonyl (Co_2_(CO)_8_). The utilization of readily accessible components obviated the need for the intricate synthesis and handling of air-sensitive cobaltate anions. Furthermore, AlPcCl has proven advantages due to its scalability, low cost, and high yield in contrast to the synthetically and economically demanding TPPCr(III)Cl. The catalyst system demonstrates exceptional efficacy not only in mono-carbonylation, but also in the generation of one-pot double-carbonylated product anhydrides, achieved through the meticulous selection of reaction conditions [57]. This innovative approach not only broadens the scope of ring-expansion carbonylation, but also emphasizes the practicality and efficiency of utilizing commercially available components for catalytic transformations, enhancing the potential for industrial-scale applications.

**Figure 7:**
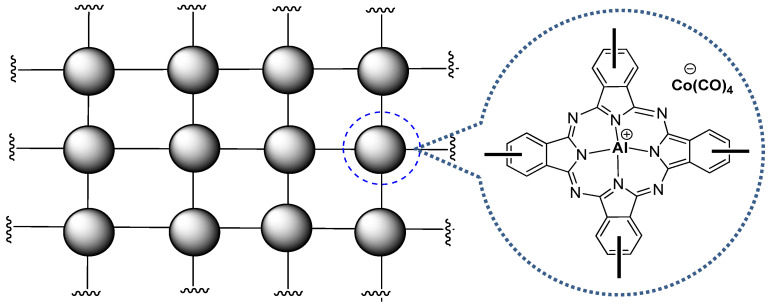
Structural representation of the picket fence type [POP-AlPc]^+^[Co(CO)_4_]^−^ heterogeneous catalyst.

The successful utilization of homogeneous aluminum phthalocyanine (AlPc) as a catalyst has spurred the development of phthalocyanine-based heterogeneous catalysts due to their outstanding activity and straightforward, cost-effective synthesis. An innovative AlPc-based heterogeneous catalyst was strategically devised, incorporating bulky substitutions on the periphery of the Pc phenyl ring to address the inherently poor solubility of phthalocyanines, typically in the range of 10^−5^ to 10^−7^ molar in common organic solvents [55]. The introduction of (2-isopropyl)-phenoxy rings effectively disrupts planar π–π stacking interactions between the macrocyclic Pc rings, leading to the formation of aggregates. The bulky substituents enhance the solubility of the Pc monomers, facilitating the construction of a novel aluminum(III)-containing picket fence-type Pc porous organic material polymer (POMP) (Figure 7).

This material was synthesized through direct knitting using aluminum chloride (AlCl_3_) catalyzed Friedel–Crafts reaction (FCR), resulting in a solid porous framework. Subsequently, this framework was employed for the incorporation of [Co(CO)_4_]^−^ anions, yielding a [Lewis acid]^+^[Co(CO)_4_]^−^-type porous heterogeneous carbonylation catalyst. Solid-state ^13^C NMR analysis of the generated polymer, compared with the monomer, revealed additional signals in the range of 43–49 ppm for the methylene linker, confirming alkylation of the aromatic ring. The chemical structure of the Pc ring remained intact during FCR. The BET surface area of the porous AlPc network and the Co(CO)_4_]^−^-incorporated catalyst was measured at 660 and 470 m^2^ g^−1^, with pore volumes of 0.6 and 0.4 cm^3^ g^−1^, respectively. ICP-AES studies and SEM-EDS analysis indicated an Al/Co ratio of 0.65, with approximately 35% of aluminum metal bound to the chloride anions. The degree of crosslinking between monomeric AlPcs hinders the replacement of Cl^−^ anions by bulky Co(CO)_4_]^−^ anions. XPS studies confirmed the successful incorporation of cobaltate anions. In the context of PO carbonylation activity, the [POP-AlPc]^+^[Co(CO)_4_]^−^ heterogeneous catalyst exhibited impressive performance at room temperature in DME solvent, with over 95% conversion of PO and greater than 99% selectivity toward β-lactone at a substrate-to-catalyst ratio of 30 (Table 2, entry 11) [55]. Comparable activity was achieved at a CO gas pressure of 10 bar, showcasing the catalyst’s robust performance across a broad substrate scope. The catalyst’s recyclability was evaluated by recovering it after the initial run and using it for successive cycles. A gradual decline in activity from the third cycle was observed, attributed to cobaltate leaching. The restoration of activity was demonstrated after regenerating the catalyst using KCo(CO)_4_. This multifaceted approach not only advances the understanding of heterogeneous catalyst design, but also underscores the versatility and efficiency of utilizing commercially available components for catalytic transformations, emphasizing its potential for scalable industrial applications.

Despite the effectiveness of the [POP-AlPc]^+^[Co(CO)_4_]^−^ catalyst in epoxide carbonylation, its β-lactone selectivity remains suboptimal. Consequently, catalysts of the [Lewis acid]^+^[Co(CO)_4_]^−^-type, featuring Lewis acidic Al^3+^ moieties, have demonstrated activity for double carbonylation, albeit yielding a mixture of products. To address this limitation and achieve both high activity and selectivity, there is a recognized need for a Cr^3+^ containing catalyst of the [PcCr(III)]^+^[Co(CO)_4_]^−^-type specifically tailored for the selective mono carbonylation of epoxides into β-lactones [56].

**Scheme 7:**
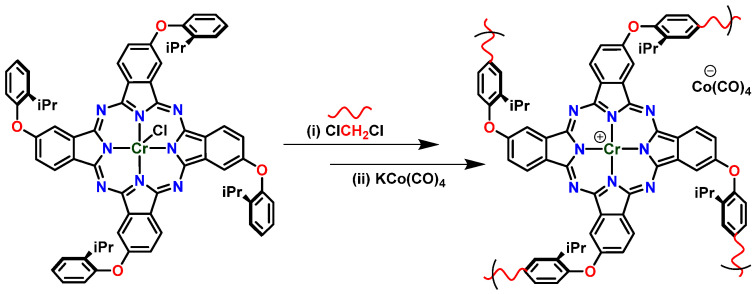
Structural representation of the synthesis of the picket fence type [POP-CrPc]^+^[Co(CO)_4_]^−^ heterogeneous catalyst [56].

In parallel with the challenges encountered with AlPc, the partial solubility of the CrPcCl metal complex arises from π–π stacking interactions. This necessitates partial structural modifications in the peripheral part of Pc to mitigate intermolecular stacking interactions and enhance the flexibility of the resulting polymeric networks. The Friedel–Crafts reaction catalyzed solvent knitting polymerization of the CrPcCl metal complex has emerged as a solution, addressing both the selectivity and flexibility concerns associated with Pc polymeric materials in the context of epoxide carbonylation. In this context, a Cr^3+^ containing Pc picket fence type POMP was strategically designed and synthesized. Functionalization with [Co(CO)_4_]^−^ anions further enhanced its utility for the carbonylation of epoxides, as depicted in Scheme 7. This innovative approach aims to overcome the solubility challenges and improve the overall performance of the catalyst in achieving selective mono carbonylation of epoxides into β-lactones [57].

In Scheme 7, the synthesis of a novel polymeric material, denoted as CrPc-POMP, is illustrated. The monomer CrPcCl was intricately assembled through aluminum chloride (AlCl_3_)-catalyzed Friedel–Crafts reaction (FCR), employing a substituted 2-isopropylphenolic group. This methodology not only enhanced the solubility of CrPcCl, but also served as a knitting group via covalent linkages facilitated by the methylene group of dichloromethane. The resultant CrPc-POMP exhibited a distinctive dark green color and exceptional robustness, displaying high porosity with a BET surface area of 725 m^2^ g^−1^ and a total pore volume of 0.388 cm^3^ g^−1^. Upon the incorporation of [Co(CO)_4_]^−^ anions through labile chlorides, a reduction in the BET surface area to 550 m^2^ g^−1^ and a decrease in pore volume to 0.28 cm^3^ g^−1^ were observed. The extensive crosslinking in the CrPc-POMP limited the complete exchange of chloride ligands, with only 38% being replaced by cobaltate anions. The remaining ligands could be confined within microporous channels or rendered inaccessible to the bulky cobaltate species.

The catalyst demonstrated remarkable activity and selectivity for the mono carbonylation of epoxides, outperforming the [AlPc-POMP]^+^[Co(CO)_4_]^−^ catalyst. Solvent-dependent activity akin to the [porphyrin ligated Cr]^+^[Co(CO)_4_]^−^ catalyst was noted, with weakly coordinating DME proving to be the most effective solvent. At 60 °C under 60 bar CO pressure, the catalyst achieved >99% conversion and >99% selectivity toward β-lactone at a substrate-to-catalyst ratio of 200 (see Table 2, Entry 12) [56].

Despite its exceptional catalytic performance, the CrPc-POMP catalyst encountered challenges in recycling, exhibiting full activity only up to the third cycle with >98% conversion in the initial two cycles. SEM-EDS analysis of the recovered catalyst supports the hypothesis of cobalt leaching as the cause of the reduced activity during recycling, a phenomenon subsequently validated by the restoration of activity after recycling [54]. While phthalocyanine-based POPs are cost-effective and amenable to synthesis, they fall short of their porphyrin counterparts in terms of activity and selectivity, as evidenced in Table 2. Additionally, akin to porphyrin-based POPs, their limited recyclability hinders their broader applicability at an industrial scale.

## 8. Conclusions

The heterogeneous ring-expansion carbonylation of epoxides for β-lactone synthesis has been reported using porous polymeric materials in handfuls. However, the catalysts developed thus far have predominantly originated from the direct heterogenization of highly active homogeneous systems or their analogs. This heterogenization approach has gained prominence due to recent advancements in various types of POPs characterized by a high specific surface area as well as excellent chemical and thermal stability. The synthesis of these POPs primarily involves Friedel–Crafts reactions, chosen for their process simplicity. The resulting catalysts exhibit a combination of homogeneous-like activity and heterogeneous characteristics, facilitating product separation and recycling.

Despite the ongoing rapid evolution of this field, future research and development should focus on several key aspects. Firstly, enhancing the current heterogenized bimetallic ring-expansive carbonylation systems is imperative for realizing economic benefits on an industrial scale. Immediate attention is required to address the reasons and mechanisms behind cobaltate leaching, aiming to improve recyclability and refine future design strategies. Additionally, the efficiency of heterogenized catalysts must be enhanced to achieve higher activity within shorter reaction times and under neat conditions. The transition from batch-type processes to continuous-flow systems should be pursued without compromising catalytic activity over prolonged durations.

## Figures and Tables

**Table 2:** Comparison among different heterogenous catalysts of carbonylation.

No.	Catalyst	-R	T °C	Solvent	Time (h)	Epoxide/Co Ratio	Yield (%)	Lactone (%)	Ketone (%)	STY h^−1^	Ref.
1	[bpy-CTF-Al(OTf)_2_]^+^[Co(CO)_4_]^−^	Me	50	DME	24	30	99	90	10	1.1	[25]
2	[Co(CO)_4_]-⊂Cr-MIL-101	n-Bu	60	DME	1	200	88	>99	-	180	[48]
3	[(POP-salph)Al]^+^[Co(CO)_4_]^−^	Me	60	DME	6	100	90	88	12	15	[51]
4	[(POP-salph)Cr]^+^[Co(CO)_4_]^−^	Me	60	DME	6	100	>99	90	10	17	[51]
5	[(PPP)Cr]^+^[Co(CO)_4_]^−^	Me	60	DME	20	1000	41	>99	<1	21	[52]
6	POP-[TPPCr]^+^[Co(CO)_4_]^−^-3h	Me	60	DME	24	2400	92	>99	<1	400 *	[53]
7	POP-[TPPCr]^+^[Co(CO)_4_]^−^-6h	Me	60	DME	24	2400	75	>99	<1	300 *	[53]
8	POP-[TPPCr]^+^[Co(CO)_4_]^−^-12h	Me	60	DME	24	2400	56	>99	<1	180 *	[53]
9	POP-[TPPCr]^+^[Co(CO)_4_]^−^-24h	Me	60	DME	24	2400	43	>99	<1	120 *	[53]
10	[HCP-(TPP)Al]^+^[Co(CO)_4_]^−^	n-Bu	60	Diox	24	1000	65	>99	<1	360 *	[54]
11	[POP-AlPc′]^+^[Co(CO)_4_]^−^	Me	60	DME	1	30	>99	>99	<1	30	[55]
12	[POP-CrPc′]^+^[Co(CO)_4_]^−^	Me	60	DME	24	200	>99	>99	<1	44 *	[56]

STY—site time yield, * STY calculated for the initial 1 h.

## Data Availability

No new data were created or analyzed in this study. Data sharing is not applicable to this article.
